# Automatic Hotspot and Sun Glint Detection in UAV Multispectral Images

**DOI:** 10.3390/s17102352

**Published:** 2017-10-15

**Authors:** Damian Ortega-Terol, David Hernandez-Lopez, Rocio Ballesteros, Diego Gonzalez-Aguilera

**Affiliations:** 1 Higher Polytechnic School of Ávila, University of Salamanca, 05003 Ávila, Spain; dortegat@gmail.com; 2 Institute for Regional Development (IDR), University of Castilla-La Mancha, Campus Universitario s/n, 02071 Albacete, Spain; david.hernandez@uclm.es; 3 Regional Centre of Water Research (CREA), University of Castilla-La Mancha, Carretera de las Peñas km 3.2, 02071 Albacete, Spain; Rocio.Ballesteros@uclm.es

**Keywords:** UAV, hotspot, sun glint, image preprocessing, photogrammetry, remote sensing, flight planning and control, software development

## Abstract

Last advances in sensors, photogrammetry and computer vision have led to high-automation levels of 3D reconstruction processes for generating dense models and multispectral orthoimages from Unmanned Aerial Vehicle (UAV) images. However, these cartographic products are sometimes blurred and degraded due to sun reflection effects which reduce the image contrast and colour fidelity in photogrammetry and the quality of radiometric values in remote sensing applications. This paper proposes an automatic approach for detecting sun reflections problems (hotspot and sun glint) in multispectral images acquired with an Unmanned Aerial Vehicle (UAV), based on a photogrammetric strategy included in a flight planning and control software developed by the authors. In particular, two main consequences are derived from the approach developed: (i) different areas of the images can be excluded since they contain sun reflection problems; (ii) the cartographic products obtained (e.g., digital terrain model, orthoimages) and the agronomical parameters computed (e.g., normalized vegetation index-NVDI) are improved since radiometric defects in pixels are not considered. Finally, an accuracy assessment was performed in order to analyse the error in the detection process, getting errors around 10 pixels for a ground sample distance (GSD) of 5 cm which is perfectly valid for agricultural applications. This error confirms that the precision in the detection of sun reflections can be guaranteed using this approach and the current low-cost UAV technology.

## 1. Introduction

Nowadays, the proliferation of unmanned aerial systems, popularly known as “Unmanned Aerial Vehicle” (UAV), is a reality for local policy makers, regulatory bodies, mapping authorities, start-ups and consolidated companies. The number of developed UAVs has increased threefold from 2005 to present and, additionally, a relevant increase has been observed in the civil/commercial type of platforms, especially in 2012 and 2013 [1]. There are many uses and benefits of UAVs based on their own pilot system (autonomous or remotely controlled) and sensory to achieve accurate positioning and to acquire a great variety of data. By this binomial, UAVs are an efficient solution for the observation, inspection, measurement and monitoring of territory; ensuring better spatial, radiometric, temporal and spectral resolution than any manned aerial vehicle and satellite platform [2]. In particular, the spectral information acquired from UAV is really important for different applications such as precision agriculture [3,4], recognition of objects [5,6,7], environmental studies [8,9] and water analysis [10]. However, the images acquired from UAVs with small-format cameras usually suffer from sun reflections problems such as sun glint or hotspot [11,12]. The sun glint effect is produced in specular surfaces such as water, glass or metal when the angle of incidence of the sun equals the angle of reflection, whereas the hotspot effect is produced due to a direct alignment between the sun, the camera and the position on the ground, that is, the point on the ground opposite the sun in relation to the camera. Both effects can generate serious problems in the modeling of terrain and in the radiometric exploitation of multispectral images. Avoid these radiometric anomalies requires of applying specific strategies for detecting these problems in UAV flight missions. At present, existing UAV software do not cope with this issue, just applying some basic preprocessing strategies based on brightness or contrast adjustment and some vignetting correction in the best case [13]. Even less, these problems are considered during flight planning and control missions. As a result, cartographic products by means of 3D models and multispectral orthoimages can lose quality and its radiometric analysis can provide wrong results in non-supervised classification procedures.

Although there are several investigations focused on image analysis for correcting similar defects on images (e.g., blur, haze, shadows), most of them are applied to satellite images [14,15]. Some approaches have been developed for aerial images acquired in manned photogrammetric flights to cope with shadows problems in mountainous landscapes [16] and even in urban areas [17,18]. Another important defect analysed in aerial images has been the blur motion [19,20,21]. However, the main problem of these aerial approaches is its high-computational cost and complexity, unfeasible when a lot of images have been acquired. In fact, some authors have analyzed the economic cost that these problems can generate in large projects [22], whereas others have put more emphasis on its scientific cost [23,24], but again applied to satellite missions. 

Regarding the topic of this paper, sun reflections problems, some authors have developed specific approaches for its removal, but most of them focused on satellite images. With respect to the sun glint effect, the principle is to estimate the glint contribution to the radiance reaching by the sensor and then subtract it from the received signal. All the methods developed are applied for satellite missions under marine environments and use to fall into two main categories: (i) open ocean imagery with spatial resolutions between 100 and 1000 m [25,26,27]; (ii) coastal images with spatial resolution less than 10 m [28,29]. A more detailed description of sun glint correction methods for satellite missions is described in [22]. However, there is not a special treatment for sun glint effects over interior water mass such as lakes, reservoirs or ponds. Furthermore, all these sun glint approaches are based on probability distributions of water surfaces and are not valid for high-resolution images acquired from UAV. Concerning hotspot effect, also known as the opposition effect or the shadow point [30], several authors have proposed different solutions: [31] propose to use homomorphic filter for removing hotspot effects in orthophoto mosaic; [32] analyse hotspot directional signatures of different agricultural crops; [33] use different kernel-driven models for analysing hotspot effects in satellite images. In [15] reflectance measurements from the spaceborne Polarization and Directionality of Earth Reflectances (POLDER) instrument are used to analyse the so-called hotspot directional signature in the backscattering direction. However, again all these developments are based on satellite images and are far from the requirements of high-resolution multispectral images acquired from UAV.

Considering the main contributions remarked above, it seems clear that up to date there is not any UAV photogrammetric software solution that copes with the detection of sun glint and hotspot effects. To this end, this paper aims to describe the algorithms developed to deal with these problems and its integration in a UAV flight planning and control software developed by the authors [34].

This paper has been structured as follows: after this introduction, Section 2 describes in detail the method developed; Section 3 outlines the new functionalities of the flight planning and control software focused on the detection of sun reflections (hotspot and sun glint); Section 4 shows and validates the method with a study case; a final section is devoted to depict the main conclusions and future lines.

## 2. Methodology

**Figure 1:**
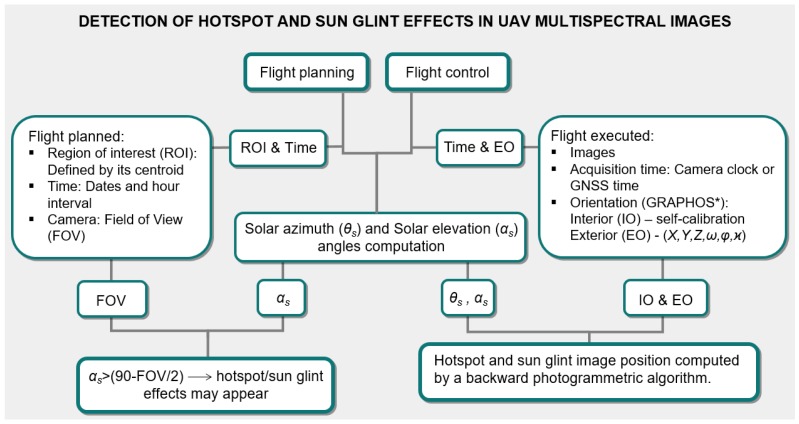
Workflow of the method developed for hotspot and sun glint detection in Unmanned Aerial Vehicle (UAV) multispectral images. * GRAPHOS (inteGRAted PHOtogrammetric Suite) is an open source software for photogrammetric applications developed by the authors.

The following figure (Figure 1) outlines the workflow developed for an automatic detection of hotspot and sun glint effects in UAV multispectral images.

Next, the main steps for the detection of sun glint and hotspot effects are described:

### 2.1. Analysis of UAV’s Metadata and Data Registration 

A specific script has been developed with a twofold function: (i) read and convert the time provided by the digital camera clock or the Global Navigation Satellite System (GNSS) solution in Universal Time Coordinated (UTC) (UTC is the bases of most radio time signals and the legal time systems. It is kept to within 0.9 s of UT (Universal Time) by introducing one second steps to its value (leap second). UT or Greenwich civil time is based on the Earth’s rotation and counted from 0-h at midnight; the unit is mean solar day. UT is the time used to calculate the solar position in the described algorithm.); (ii) register the approximate position of the GNSS from the instant acquisition time of each image using the Exchangeable image file (Exif) configuration as input data. Camera clock is the quartz clock of digital cameras which is used for establishing the date and time acquisition for images in the local time of the zone. There is not a specific format requirement and the information is read from the Exif format. One of the most important aspect of these metadata is the synchronization between the time of capture and the time of registration for both sensors (camera clock or GNSS). In our approach we have obtained differences of 200 ms. This value is insignificant for our purpose, since an error of 2 s would correspond to a displacement of 0.5 pixels for the detection of sun reflections effects.

### 2.2. Images Orientation 

Once we have solved the acquisition time with the camera clock or the GNSS, as well as an approximate GNSS position of each image, GRAPHOS [35], an open source photogrammetric software, was used for the orientation of images. GRAPHOS uses an internal geodetic local space rectangular (LSR) system (In this LSR system, the *Z* axis represents the zenith direction, the *X* axis the east direction and the *Y* axis completes a right handed system pointing to the north. The origin of this system has been defined as anchor point using the barycentre of the different images), which avoids the problems of dealing with scale and absolute coordinates provided by the GNSS solution. Absolute coordinates deal with two different reference systems: one for planimetric coordinates (usually, an ellipsoid with cartographic UTM projection); and other for altimetric coordinates (usually, a geoid with orthometric altitudes). Since both coordinates are supported by different reference systems, the scale definition will be different along the planimetric and altimetric axes. Furthermore, those problems related with coordinate reference systems such as the passing from terrain to ellipsoid and the passing from the ellipsoid to Universal Transverse Mercator (UTM) projection can be solved with higher precision in the orientation of images and thus in the photogrammetric products obtained. 

It should be remarked that this internal geodetic LSR approach is not considered in any of the most well-known commercial image-based modeling software, being really useful when we deal with different sensors (e.g., GNSS, multispectral camera, etc.).

Images orientation is solved through a combination between computer vision and photogrammetry. First, this combination is fed by the resulting keypoints extracted and matched using Maximal Self Dissimilarity (MSD) [36] and Scale Invariant Feature Transform (SIFT) [37] algorithms, both included in GRAPHOS. In particular, keypoints are detected with MSD and described with SIFT. The descriptor is mandatory to assign attributes to the point and thus for performing the matching (search of homologous points) between images. Next, an approximation of the external orientation of the cameras is calculated following a fundamental matrix approach [38]. The external orientation of images include the spatial (*X*, *Y*, *Z*) and angular (omega, phi y kappa) positions. Finally, these positions are refined by a bundle adjustment complemented with the collinearity condition based on the Gauss–Newton method [39], obtaining the final external orientation of images. GRAPHOS solves the external orientation based on the combination of two open source solutions such as Bundler [40] and Apero [13]. In addition, the external orientation of images allows to integrate as unknowns several internal camera parameters (i.e., focal length, principal point and lens distortions), allowing the use of non-calibrated cameras and guarantying acceptable results. For the present study case, a self-calibration strategy supported by Fraser calibration model which encloses seven internal parameters (focal length, principal point, two radial distortion parameters and two tangential distortion parameters) is used [41]. This final orientation can be performed with internal constraints (i.e., free-network) or with external constraints (e.g., Ground Control Points—GCPs or known distances) using the collinearity condition (Equation (1)):

(1) $$\begin{array}{l} (x-x_{0})+\Delta x=-f\frac{r_{11}(X-S_{X})+r_{21}(Y-S_{Y})+r_{31}(Z-S_{Z})}{r_{13}(X-S_{X})+r_{23}(Y-S_{Y})+r_{33}(Z-S_{Z})}\\ (y-y_{0})+\Delta y=-f\frac{r_{12}(X-S_{X})+r_{22}(Y-S_{Y})+r_{32}(Z-S_{Z})}{r_{13}(X-S_{X})+r_{23}(Y-S_{Y})+r_{33}(Z-S_{Z})} \end{array}$$

where *x* and *y* are the known image coordinates coming from the matching of keypoints; *X_i_*, *Y_i_* and *Z_i_* are the corresponding known GCPs or known distances coming from topographic surveying or existing cartography; *r_ij_* are the unknown 3 × 3 rotation matrix elements; *S_X_*, *S_Y_* and *S_Z_* represent the unknown camera position; *f* is the principal distance or focal length; *x*_0_ and *y*_0_ are the principal point coordinates and ∆*x* and ∆*y* are the lens distortion parameters. These internal camera parameters may be known or unknown by the user and thus are introduced as equations or unknowns (self-calibration), respectively. This equation is not linear and has to be solved iteratively based on initial approximations.

It should be noted that in those cases where the UAV can use a GNSS with enough quality (phase solution with monofrequency L1 or bifrequency L1–L2), direct orientation of images can be solved and thus no GCPs would be required.

### 2.3. Solar Positioning 

Once images have been oriented with a robust photogrammetric approach, the relative positioning of the sun (solar azimuth and elevation) is computed based on the UTC time and using geodetic coordinates (latitude, longitude and ellipsoidal altitude) of each image. In particular, solar azimuth (*θ_S_*) and solar elevation (*α_S_*) angles are computed for each image using the following equations (Equations (2) and (3)). In this step the open source solar library SPA is used (http://rredc.nrel.gov/solar/codesandalgorithms/spa/).

(2) $$\theta_{S}=\Theta +\Delta \psi +\Delta \tau$$

where *θ_S_* is the solar azimuth angle, ∆*τ* is the aberration correction (in degrees), ∆*ψ* is the nutation in longitude (in degrees) and Θ is the geocentric (Geocentric means that the sun position is calculated with respect to the Earth center) longitude (in degrees).

(3) $$\alpha_{s}=\arctan\;2\left(\frac{\sin\;\theta_{S}\times \cos\;\varepsilon -\tan\;\beta \times \sin\;\varepsilon }{\cos\;\lambda s}\right)$$

where *α_S_* is the solar elevation angle (in radians), *θ_S_* is the solar azimuth angle, *ε* is the true obliquity of the ecliptic (in degrees) and *β* is the geocentric latitude (in degrees).

### 2.4. Hotspot and Sun Glint Detection 

**Figure 2:**
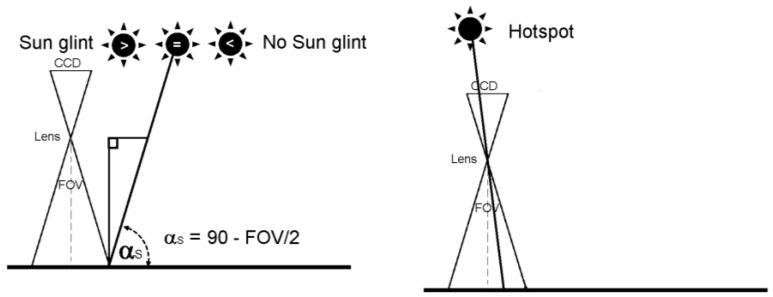
(**Left**) *Sun glint effect*: the sun glint effect is produced from specular surfaces such as water, glass or metal when the angle of incidence of the sun equals the angle of reflection and this angle is lower than the field of view (FOV) of the camera. In particular, if the sun elevation (*α_S_*) is greater than 90 minus 1/2 the FOV of the camera, rays from the sun can be reflected directly onto the charge-coupled device (CCD) causing a sun glint defect on the photo; (**Right**) *Hotspot effect*: the hotspot is produced due to a direct alignment between the sun, the camera and the position on the ground, that is, the point on the ground opposite the sun in relation to the camera. The hotspot produces a change of brightness in this point of the image and its surroundings.

Computed the relative solar positioning (azimuth and elevation solar angles) for each image, the theoretical position of the possible hotspot and sun glint effects is provided for each image following the next photogrammetric steps. Figure 2 outlines both effects and its geometrical basis.

- Determination of hotspot/sun glint direction angles (azimuth and elevation). In the case of hotspot, the azimuth angle (*θ_hs_*) is computed as the solar azimuth *θ_S_* ± 180 degrees. In the case of sun glint, the azimuth angle (*θ_sg_*) is computed using directly the solar azimuth (*θ_S_*). The elevation for both effects (*α_hs_*, *α_sg_*) corresponds with the relative sun elevation for each image (*α_S_*).
- Transformation between coordinate reference systems. In order to guarantee better accuracy in the photogrammetric process, a transformation from geodesic coordinates (latitude, longitude and ellipsoidal altitude) to LSR coordinates is performed for each image (see Section 2.2).
- Hotspot/sun glint direction vector. Using an arbitrary distance (e.g., 150 m), the sun elevation and azimuth angles (*α_S_*, *θ_S_*) and the image orientation in LSR coordinates, a vector is defined in the internal geodetic LSR system for each image. This arbitrary distance is defined with the length of a vector with origin in the projection center of the camera (*S_X_*, *S_Y_*, *S_Z_*) and with the direction of the optical axis of the camera (*r_ij_*). Flight height can be a reference for establishing the length of this arbitrary distance. As a result, a direction vector for the possible hotspot (*θ_hs_*, *α_hs_*) and sun glint (*θ_sg_*, *α_sg_*) effect is defined. It should be noted, that all the points of the vector are projected in the same image point, so the distance chosen is completely arbitrary.
- Hotspot/sun glint ground coordinates. Using the hotspot/sun glint direction vector the hotspot/sun glint ground coordinates (*X*, *Y*, *Z_hs_*_/*sg*_) are computed in the internal geodetic LSR system.
- Hotspot/sun glint image coordinates. A backward photogrammetric process is applied to detect both effects in the images, using the external and internal orientation of each image and based on the collinearity condition (Equation (1)). If the image coordinates computed (*x*, *y_hs_*_/*sg*_) (in pixels) are within the format of the camera, hotspot or sun glint effects will appear in the images.
- Masks definition. Since all the process developed accumulate errors (e.g., from the acquisition time to the inner and exterior orientation parameters) and the own effects enclose a size, a buffer area definition of 50 × 50 pixels is defined around these hotspot and sun glint coordinates to isolate those parts that can be affected by sun effects in the images. This buffer size is related with the ground sample distance (GSD) or pixel size. In our case, the GSD is 5 cm, so a buffer area of 50 × 50 pixels corresponds to a ground area of 2.5 m × 2.5 m. Since the sun glint and hotspot effects can show different sizes and shapes, this value is considered enough for enclosing the effect, including also the own propagation error of the process. Masks were used in the photogrammetric process as exclusion areas, that is, nor keypoints used in the orientation of images, neither the image points sampled during the orthophoto generation were taken from these exclusion areas. Alternatively, these exclusion areas were completed taking advantage of the high number of images acquired in UAV flights and the high overlap between images (70%).

## 3. UAV Flight Planning and Control Software: MFlip-v2

A specific module for dealing with hotspot and sun glint effects has been added to the software MFliP-v1 [34], which can be used as an interface applicable to a wide variety of UAVs. The application was implemented using the C++ programming language and Extensible Markup Language (XML) for data management. This section provides an overview of the specific module for detecting hotspot and sun glint effects. It should be noted that sometimes the flight must be planned when the sun provides maximum irradiance to the crops (e.g., analysis of crop water stress), so there is more probability to suffer these effects. Although under these circumstances the flight cannot be changed, it will be very useful to detect these problems in order to isolate them in the photogrammetric or remote sensing processing.

**Figure 3:**
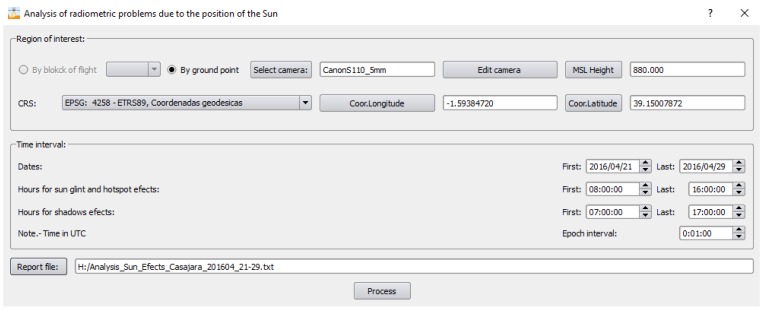
Layout of the developed software, MFliP-v2, for hotspot and sun glint detection in UAV flights.

Figure 3 shows a screen capture of the software application and its specific module for hotspot and sun glint effects. In this overview we show a simple case of use step-by-step focused on detecting hotspot and sun glint effects during flight planning mission. 

### 3.1. Project Definition 

When users run the program, they first create a new project. The user is asked to fill in a form with some necessary data for the program to work. All this information is stored in database. This data describes the settings of the UAV flight: coordinate system, region of interest (ROI), flight direction and the date and time (in UTC) of the flight. In addition to the settings of the flight, the settings of the camera must also be entered into the form. 

**Figure 4:**
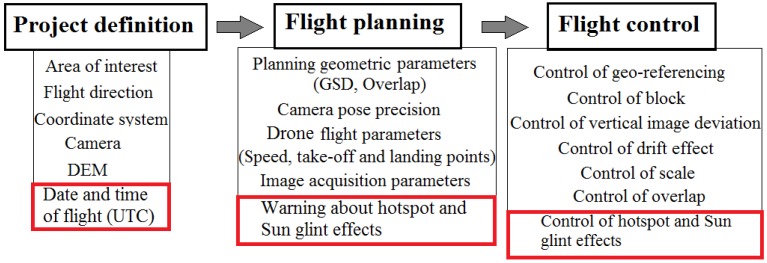
Flowchart for flight planning and control with special attention to hotspot and sun glint effects (highlighted in red colour). The rest of functions in flight planning and control can be analysed in detail in [34].

In the following figure (Figure 4) the different steps from project definition to flight planning and control are outlined, highlighting (in red colour) the new module for dealing with hotspot and sun glint effects.

### 3.2. Flight Planning 

Once we have an active project, we must plan the flight. The first step is to delimitate the ROI together with the flight direction as well as the date and time of the flight. Based on the centroid coordinates of the ROI and the theoretical position of the planned images along the flight direction, a planned sun position (azimuth and elevation) for each image are computed. If the sun elevation angle (*α_s_*) is greater than 90 minus 1/2 the FOV of the camera (Figure 1), the software will alert about the possible presence of hotspot and sun glint effects to the user, giving information about the time interval in which we can suffer these problems. 

As a result, a flight planning file is automatically generated by the program and directly uploaded to the UAV firmware.

### 3.3. Flight Control

Once the flight was executed, the control of the flight is performed based on the validation of different geometric controls: geo-referencing, vertical image deviation, drift effect, scale and overlap. More detailed information about these geometric controls can be found in [34]. Regarding hotspot and sun glint effects, during the control step both effects are detected in those images where exist (see Section 2) and then are isolated with a mask for the next photogrammetric and remote sensing processes. 

## 4. Experimental Results

**Figure 5:**
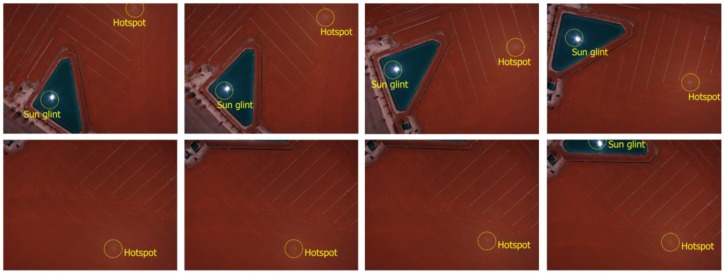
Hotspot and sun glint effects in the region of interest. It should be noted how the position of both effects varies through the different images.

In this section we check the algorithms developed using a UAV flight executed with a multispectral camera. The area over which images were acquired covered an area of 820,000 m^2^ with an approximately rectangular shape of 1050 m × 780 m. The site was located in Tarazona de la Mancha (Albacete, Spain). The main reason to perform this test was the application of this methodology to photogrammetric and remote sensing applications. In particular, a near infrared orthophoto and normalized digital vegetation index (NDVI) were computed for agronomical studies. The following figure (Figure 5) outlines the main problems of hotspot and sun glint effects over the region of interest. 

Regarding the platforms and sensor used, a Carabo S3 UAV was used (Table 1) together with a Canon PowerShot S110 compact digital camera. This camera allows us to include a specific near infrared filter [42], recording near infrared, green and blue channels, at the same time. The specific operational conditions (Table 1) were integrated through the autopilot-flight control based on Pixhawk with a processor (168 MHz/252 MIPS Cortex-M4F) and a firmware (APC Copter 3.4.6). Regarding Canon PowerShot S110 and its synchronization, an electronic switch connected to the multiport entry through a micro-USB connector was used. This switch is activated by one of the outputs of the Pixhawk autopilot based on the commands included in the flight mission. In addition, Cannon incorporates a particular firmware, CHDK, which allows us to shoot camera remotely.

Last but not least, the position and time provided by the GPS are stored in a log file and inserted in the heading of each image. This operation is performed using one of the tools provided by the flight planning (Geotag), which receives as input the own images and the log file remarked before, writing in the exif file of each image the approximate coordinates and GPS time. It should be noted that this procedure is valid for any UAV based on the same autopilot (Pixhawk).

**Figure 6:**
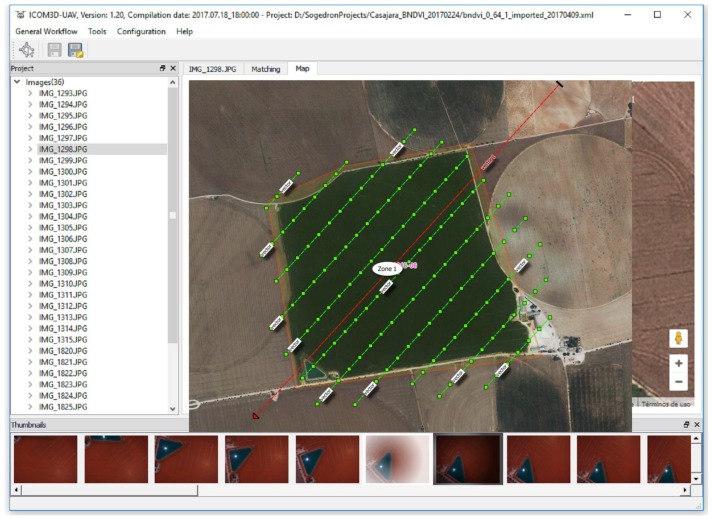
Flight planning over the region of interest (ROI). This planning was defined using the centroid *X*, *Y*, *Z* coordinates of the ROI together with the flight direction and the date/time of the flight.

The flight was planned using the region of interest, the flight direction and the date/time interval as input (Figure 6). In addition, aspects such as the GSD, flight height, flight speed, waypoints and overlap between adjacent images were also considered. Particularly, a GSD of 5 cm which corresponds to a flight height of 140 m was considered. Overlaps between images of 70% and 25% were planned for forward and side overlaps, respectively. Regarding the flight speed, two flight speeds were considered: (i) a cruising speed usually of 6 m/s and (ii) an acquisition speed close to 0 m/s when the UAV is in the shooting point. This last issue is crucial in order to minimize the inertial effects of the own UAV movement. Since the precision of navigational sensors (GPS, accelerometers, gyroscope, etc.) is not high, better results will be obtained if images are acquired with the UAV stable and without too much speed. Regarding the shooting rate of the camera, it was setup according to the waypoints of the flight planning. Therefore, the camera acquired images when it is in the waypoint planned, both for planimetric coordinates (*X*, *Y*) and altimetric coordinates (*Z*). As result, we can guarantee the overlaps, scale and the different geometrical constraints for the flight planned. Finally, the camera setup parameters were fixed before the flight. In particular, in our flight these setup parameters were: manual focus fixed to infinity, exposure time: 1/1000, aperture of diaphragm: ±5.6 and automatic ISO. 

**Figure 7:**
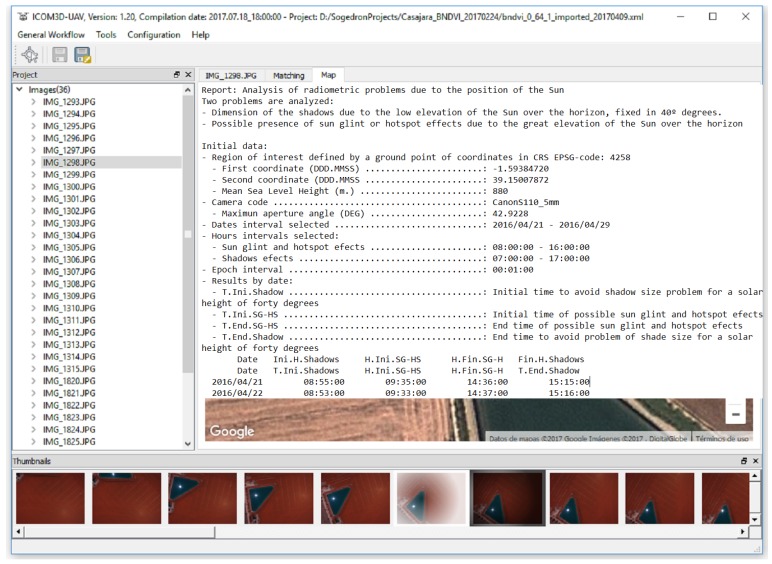
Detailed report about the interval time for possible hotspot/sun glint effects. Note that the time interval for sun glint and hotspot effects are broad since we are using a digital camera with a large field of view.

On the other hand, a previous analysis of sun reflections was performed during flight planning using the field of view (FOV) of the camera and the planned sun elevation angle. In particular, if the sun elevation angle (*α_S_*) is greater than 90 minus 1/2 the FOV of the camera, hotspots and/or sun glint effects may appear. A detailed report about the interval time (in UTC) for both effects is provided, warning the user about possible hotspot and sun glint effects and their time intervals (Figure 7).

Since it was required to fly at these time intervals due to agronomical reasons, a control of the flight was mandatory to detect possible sun reflections problems. To this end, a proper interior and exterior orientation of the camera is crucial in order to guarantee quality in the detection of hotspot and sun glint effects and thus accurate products in photogrammetry and remote sensing applications. 

In our case, the Canon PowerShot s110 digital camera (Canon Inc., Tokyo, Japan) was calibrated based on a bundle adjustment using the self-calibration approach included in GRAPHOS software. The self-calibration results and other camera parameters are shown in Table 2. The orientation information of the images was computed using the keypoints extracted and matched and the GCPs. In particular, these GCPs were extracted from public cartographic products (In Spain, the National Geographical Institute (IGN) periodically provides free cartographic products by means of orthoimages and LiDAR flights. Both products belong to a National Program for Earth Observation (PNOT) supported by the directive INSPIRE (Infrastructure for Spatial Information in Europe). More information can be found in: http://www.ign.es/web/ign/portal/obs-area-observacion-territorio.): *X*, *Y* coordinates coming from a public orthophoto (pixel size of 25 cm) and *Z* coordinate coming from a public Light Detection and Ranging (LiDAR) flight (resolution 0.5 points/m^2^). The accuracy for keypoints was subpixel, the accuracy for GCPs was 0.5 m and the accuracy for orientation was 0.2°. 

**Figure 8:**
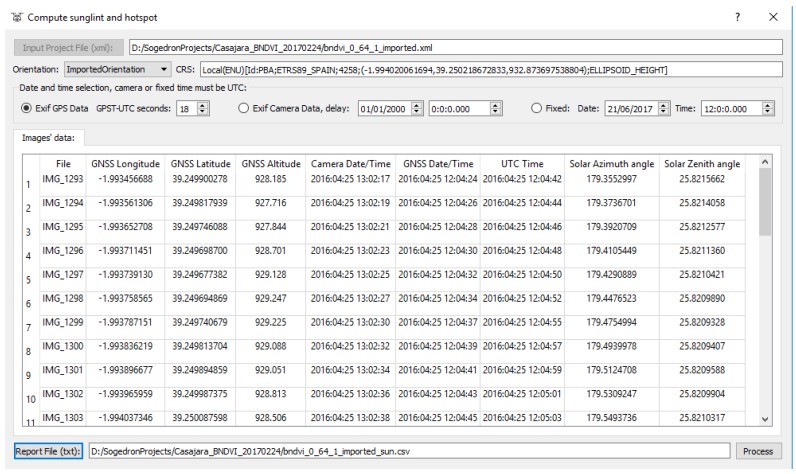
Solar positioning (azimuth and elevation) for the study case analyzed.

Once images were oriented using the self-calibration approach, the relative solar positioning (sun azimuth-*θ_S_* and sun elevation-*α_S_*) was computed for each image. Figure 8 shows the relative solar positioning (sun azimuth-*θ_S_*, and sun elevation-*α_S_*) for each image based on the camera clock or GNSS time, as well as the orientation for each image.

Later, hotspot and sun glint effects were detected (in pixel coordinates) based on a backward photogrammetric approach (Section 2.4) using the relative position of sun and the photogrammetric orientation of the images. If these coordinates are within the camera format the effects are detected and isolated with a specific mask. Table 3 shows the main results in pixel coordinates.

Last but not least, considering the heterogeneity of the precision of input data, a simulation study for analyzing the a priori precision based on an error analysis was performed using the following simulated errors:

- GNSS positioning errors (meters): 0.1, 0.2, 0.5, 1, 2, 5, 10, 20, 50, 100;
- Time errors (seconds): 0.1, 0.2, 0.5, 1, 2, 5, 10, 20, 50, 100;
- Orientation (degrees): 0.05, 0.1, 0.2, 0.5, 1, 2, 5, 10, 20.

Positive and negative errors were considered for each parameter.

**Figure 9:**
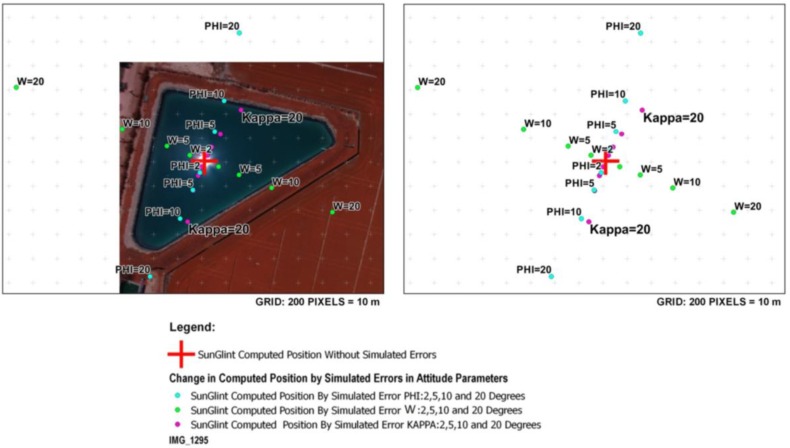
Analysis of errors for the detection of sun glint effects simulating different errors in angular position (omega, phi, kappa).

The following figure (Figure 9) outlines the results of this simulation of errors. It is important to note that only those very high errors coming from the orientation parameters have representation at this scale. 

From the analysis of Figure 9 we can see that an error of 2° in phi or omega angles causes a variation in the position of 100 pixels; an error of 2° in kappa angle causes a variation in the detection of 50 pixels. Regarding time, although the representation scale does not allow us to visualize it, an error of 2 s in time would cause a variation in the detection of 0.5 pixels. This time error of 2 s does not take part in the computation of the orientation parameters of the images. Instead, this orientation of images (spatial and angular) is solved based on photogrammetry with collinearity condition and the GCPs, as was explained in Section 2.2. As a result, the error of 2 s which provides a variation of 0.5 pixels is related with the Sun position. Therefore, a delay of 2 s in the variation of sun position is insignificant regarding the sun reflection effect in the images. Regarding GNSS, although the representation scale does not allow us to visualize it, an error of 30 m in the position (latitude, longitude y altitude), would provide variations of detection lower than 0.01 pixels.

The performed analysis of the errors aims to highlight two main issues:

(a) The main errors in detection of sun reflections are related with the camera orientation parameters. Therefore, it is crucial to apply a photogrammetric approach to solve this orientation (Section 2.2.).
(b) Simulated spots correspond to errors in orientation which are far from the errors obtained in our photogrammetric approach, even when low-cost UAV sensors are used.

Therefore, this error analysis supports that the precision in the detection of sun reflections is enough and is guaranteed using this photogrammetric approach and the low-cost UAV technology.

In our case of study, an error in the georeferencing based on GCPs of 0.5 m provides subpixel variations, an error for the orientation of 0.2° provides a variation of 10 pixels and a time error of 1 s provides a variation of 0.25 pixels. As a result, an error of approximately 10 pixels can be considered for the detection of sun glint and hotspot effects. Therefore, the most relevant conclusion about this error analysis is that the orientation of images is the most important step for the detection of sun reflections effects, so the photogrammetric approach is crucial.

**Figure 10:**
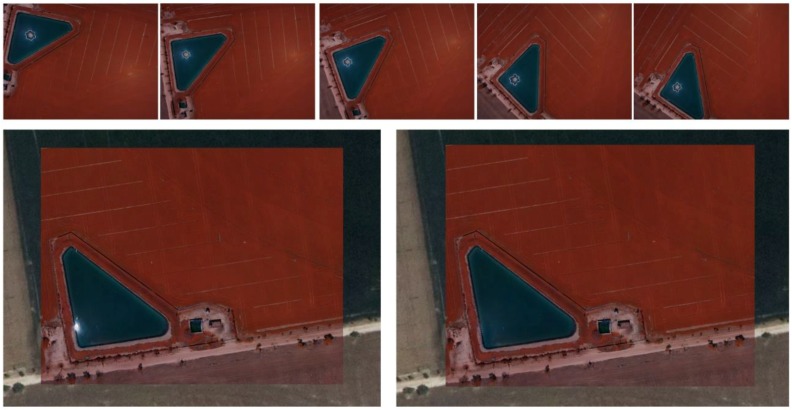
Near infrared orthophoto. (**Up**) detection and clustering with a mask of sun glint effects over near infrared images; (**Down**) comparison between resulting near infrared orthophoto with sun glint correction (**right**) and without sun glint correction (**left**).

**Figure 11:**
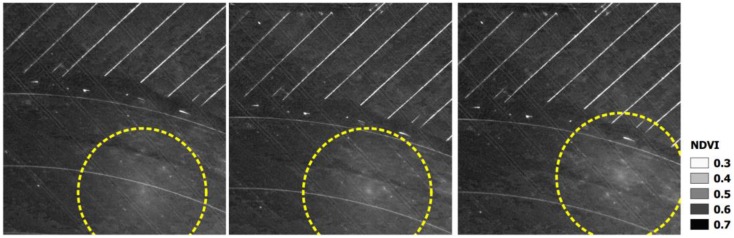
Normalized digital vegetation index (NDVI) images and the corresponding NDVI values in the hotspot areas detected (yellow circle).

Finally, a near infrared (NIR) orthophoto (Figure 10) and NDVI images (Figure 11) were computed once the different sun reflection effects were detected and excluded. Note the difference between the NIR orthophoto without applying the process (Figure 10 left down) and the NIR orthophoto detecting and correcting sun reflections effects (Figure 10 right down). Regarding NDVI images, these images were computed using the custom vegetation-sensing filter included in this camera by the manufacturer (https://event38.com/product/custom-ngb-filter-glass-for-diy-camera-conversion/). This filter allows an off-the-shelf camera to be used to collect NIR, Green and Blue channels. With this data, a pseudo NDVI can be calculated as follows (Equation (4)):

(4) $$pNVDI=\frac{NIR-Blue}{NIR+Blue}$$

Figure 11 shows how affects the hotspot effect to the pseudo NDVI values in a qualitative and quantitative (scale bar) way. Particularly, differences higher than 20% were obtained for pseudo NDVI in those hotspot areas where pseudo NVDI values clearly decrease and whose values could directly affect the different agronomical analysis (e.g., determination of irrigation crops). Figure 11 describes that the same area on the ground can show different values of NDVI, even for those images taken under very low time lapse and very close point of views due to the hotspot effect. That means that this portion of the image under hotspot effects should not be used for vegetation indices estimation, especially if other images which show this area are not under hotspot effects.

## 5. Conclusions

Through this article has been confirmed the necessity and importance of developing procedures that automate the detection of solar reflection problems in high resolution images acquired from UAV. In fact, currently there is not any commercial or scientific solution in the field of UAV that can deal with this problem, which could have serious consequences both in photogrammetric and remote sensing processes, especially if there is a large number of images. The process developed allows to solve the problem from a twofold perspective: from its possible prevention during flight planning to its detection once the flight was executed applying a flight control protocol, especially for those situations in which is mandatory to fly during the hours where the sun reaches its maximum height, being inevitable that these defects can appear.

The developed process has been implemented in a software for planning and control of UAV flights previously developed by the authors [34], allowing an easy handling of this type of problems. 

The experimental results have showed the efficiency of the method to obtain photogrammetric products of higher quality and lower noise (e.g., near infrared orthophoto), as well as to obtain a much more accurate normalized vegetation indexes (NVDI) which are crucial in agronomical analysis. The error analysis performed aims to outline two main aspects: (i) the main errors that could affect the spots detected are related with the camera orientation parameters; (ii) the precision in the detection of sun reflections is around 10 pixels, which is guaranteed with our photogrammetric approach and using the current low-cost UAV technology.

The line developed so far opens new possibilities of improvement associated to the own method and focused on being able to implement strategies that allow not only to detect and isolate the sun reflection problems but also to correct these areas using other acquired images. Furthermore, since sun glint can only occur on reflective surfaces, it would be interesting to develop a strategy that allows to supervise automatically those areas which could not contain reflective surfaces without user intervention.

## Figures and Tables

**Table 1:** Technical specifications and operational conditions: UAV Carabo S3.

**Technical Specifications: UAV Carabo S3**	
Climb rate	2.5 m/s
Cruising speed	6.0 m/s
Vehicle mass	2.6 kg
Recommended payload mass	450 g
Maximum payload mass	900 g
Dimensions	690 mm from rotor shaft to rotor shaft
Flight duration	up to 40 min (Full HD smart sport camera)
Battery	6 S/22.2 V
Accelerometer/magnetometer	ST Micro LSM303D 14 bit
Accelerometer/gyroscope	Invensense MPU 6000
Gyroscope	ST Micro L3GD20H 16 bit
Barometer	MEAS MS5611
GPS	uBlox LEA 6H
Autopilot	Pixhawk
**Operational Conditions: UAV Carabo S3**	
Temperature	−10 °C to 45 °C
Humidity	max. 90% (rain or snow is no problem)
Wind of tolerance	up to 12 m/s
Flight radius	500 m on RC (Remote Control), with WP (Way Point navigation) up to 2 km
Ceiling altitude	up to 2500 m

**Table 2:** Technical specifications and camera self-calibration parameters: Canon Powershot S110.

**Technical Specifications: Canon Powershot S110**	
Sensor Type	1/1.7” CMOS sensor
Sensor size (*w*, *h*)	(7.2, 5.4) mm
Effective Pixels	4000 × 3000 pixels
Focal length	5.2 mm
Weight	198 g (inc battery)
**Self-Calibration Parameters: Canon Powershot S110**	
Calibrated focal length	5.054 mm
Principal point (*x*, *y*)	(−0.034, −0.126) mm
Radial distortion (*k*_1_, *k*_2_)	(−0.0370017, −0.00429136)
Tangential distortion (*P*_1_, *P*_2_)	(−0.00116555, −0.00518746)

**Table 3:** Example of hotspot and sun glint detection in image coordinates.

Image	Sun Glint Detection (*x, y* in Pixels)	Hotspot Detection (*x, y* in Pixels)
IMG_1293	n.a.	(2804, 2327)
IMG_1294	(1160, 16)	(2833, 2355)
………......	……………….	……………….
IMG_1303	(1153, 2215)	n.a.
IMG_1304	(1098, 2243)	(2962, 47)
IMG_1305	(1109, 2237)	(2960, 26)
…………..	……………..…	……………..…
